# Inflammatory Proteomic Network Analysis of Statin-treated and Lipopolysaccharide-activated Macrophages

**DOI:** 10.1038/s41598-017-18533-1

**Published:** 2018-01-09

**Authors:** Abu Hena M. Kamal, Jayanta K. Chakrabarty, S. M. Nashir Udden, Md. Hasan Zaki, Saiful M. Chowdhury

**Affiliations:** 1 Department of Chemistry and Biochemistry, University of Texas at Arlington, TX, 76019 USA; 2 Department of Pathology, UT Southwestern Medical Center, Dallas, TX 75390 USA

## Abstract

A significant component of immune biology research is the investigation of protein encoding genes that play central roles in contributing inflammatory response. A gel-free quantitative bottom-up proteomics study was performed on immune cell macrophages after the combined treatment of lipopolysaccharide (LPS) and statin drugs using mass spectrometry and a detailed bioinformatics analyses were conducted. Systematic bioinformatics analysis was applied for discovering novel relationships among proteins and effects of statin and lipopolysaccharide in macrophage cells. Based on gene ontology, majority of protein encoding genes was involved in metabolic and cellular processes and are actively associated with binding, structural molecular, and catalytic activity. Notably, proteomic data analyzed by Ingenuity Pathway Analysis (IPA), discovered the plectin and prohibitin 2 protein interactions network and inflammatory-disease based protein networks. Two up-regulated proteins, plectin and prohibitin 2, were further validated by immunoblotting. Plectin was also cross-validated by immunocytochemistry, since its expression was highly modulated by statin but inhibited during LPS-stimulation. Collectively, the significant up-regulation of plectin due to the treatment of statin, suggests that statin has a significant impact on the cytoskeletal networks of cells. Plectin might have a significant role in the intermediate filament assembly and dynamics, and possibly stabilizing and crosslinking intermediate filament networks.

## Introduction

Macrophages are immune cells, which are found in different tissues and perform a broad range of cellular functions^1,2^. Macrophages play major roles in host defense, inflammatory responses, and immunity. They are activated by wide range of cytokines and microbial ligands and produces immune response by activating Toll-like-receptors (TLRs) signaling pathways^3^. When macrophage cells are stimulated and activated by microbial and viral ligands, they produce proinflammatory cytokines, nitrogen species, and oxygen^1^. These cytokines were employing the inflammation to the site of the infection, thereby intensifying and enabling the inflammatory conditions^4^. Lipopolysaccharide (LPS) in bacteria or together with cytokines such as interferon-gamma (IFNγ) are well-known stimulation agents for inducing the transcription of genes during any kind of proinflammation in macrophages^5^. When Raw 264.7 macrophage cells are stimulated by LPS, TLR4 signaling pathways get activated, which in turn activates transcription factor nuclear factor-kB (NFkB) and secretes different kind of cytokines such as tumor necrosis factor-α, IL1-beta, and IL-6^6^. In response to host-defense, the cell increases the expression of cyclooxygenase 2 and nitric oxide (NO) synthase^7^, and releases prostaglandin E2^8,9^ and NO^10,11^ which further intensify the inflammation.

Statins are commonly prescribed medicines for managing hypercholesterolemia. Statins are aggressive and competitive suppressors of 3-hydroxy-3-methylglutaryl coenzyme A (HMG-CoA) reductase, and act as catalyst to the rate-determining step in the biosynthesis of cholesterol. They reduce the occurrence of cardiovascular diseases by lowering low-density lipoprotein (LDL) and triglyceride levels^12^. Statins control the production of mevalonate at the cellular level that is a precursor of nonsterol compounds, which play a vital role in cellular functions. Moreover, several studies have revealed that mevalonate-derived isoprenoid substrates, farnesyl pyrophosphate (FPP) and geranylgeranyl pyrophosphate (GGPP), are the precursors for posttranslational protein modifications prenylation^13,14^. Statins may possibly modulate the process of inflammation^15^ that have been reported to develop endothelial dysfunction, direct the thrombosis, boost the solidity of atherosclerotic plaques, and reduce oxidative stress^16^. These pleiotropic possessions, while involving the useful effects on cardiovascular disease, also appear to play a vital responsibility in further inflammatory and immune diseases. Statins showed to decrease the risk allied to sepsis, including disease progression, mortality, and incidence in human clinical trials^17–20^.

Based on previous studies, LPS acted as a key facilitator in the inflammatory stage of many diseases, and statins are widely used lipid-lowering drugs for humans. Proteomics are widely accepted approaches to investigate differential protein expression, which also helped to reveal the cellular and biological mechanisms at the molecular level in cells^21,22^. A number of quantitative proteomics studies have been reported on the treatment of LPS on macrophage cells. Label-free proteomics study was performed on LPS-treated human monocytes (THP-1 cell line) to understand the differentiation and activation of monocytes in pro-inflammatory states. This study suggested LPS-treated monocytes could potentially be used for tumor therapeutic applications^23^. LPS induced study on raw macrophage cells were conducted using two-dimensional (2D) gel electrophoresis and mass spectrometry. Eleven proteins were quantified by mass spectrometry due to their differential expressions in gel spots^24^. Stable isotope labeling with amino acid (SILAC) based quantitative proteomics studies on LPS stimulated macrophages were done on cytosolic and nuclear fractions. Upon 10 min LPS exposures, several key proteins were modulated which were found related to mitogen-activated protein kinase (MAPK) and NF-kB signaling pathways. This study provided first system-wide insight and cross-talk between signaling pathways and transcription factors and subsequent activation of pro-inflammatory genes^25^. Quantitative proteomics study was also performed on lipid rafts isolated from LPS treated raw macrophages^26^. Chowdhury *et al*.^22^ identified 383 unique proteins in isolated raft of LPS-stimulated ABCA1-deficient primary mouse macrophage cells. Proteomic study on microtubule (MT)-associated proteins in activated macrophages by LPS and interferon-γ (IFNγ), whereas 409 MT-associated protein were identified in MT purified samples^27^. Shi *et al*.^28^ performed proteomic investigation on *Salmonella*-infected macrophage cells in a time-dependent manner. More than 1000 proteins were identified, and 244 proteins were significantly altered after infection. Swearingen *et al*.^29^ used Isotope Coded Affinity Tagging (ICAT) to profiling the LPS-induced proteins in Raw 264.7 macrophage cells. In more than 1000 proteins, 36 proteins were significantly changed their differential expression with LPS-stimulation.

Study on primary macrophages by loading and unloading cholesterols was performed using mass spectrometry. Subsequent study was also performed by cells deficient of low density lipoprotein receptor (LDLR) and apo lipoprotein E to understand the protein network in *Atheroscelerosis* ^30^. A small clinical study was performed with healthy individual treated with rosuvastatin. Label free spectral counting approach on lipoprotein fractions (high density-lipoprotein, HDL-S/L and LDL) found significant changes on lipoproteins pools and abundance of protein alpha 1-antirypsin in HDL-L fractions^31^. In addition, a study was performed on the global profiling of sialic acid status on macrophages upon treatment of statin. Moreover, Gorden *et al*.^31^ performed lipoprotein proteomics in human blood sample to understand the effect of statin (rosuvastatin) in immune system, where 154 proteins were identified.

As mentioned above, several proteomics studies were reported in Raw 264.7 macrophage cells utilizing LPS as well as with pathogenic microbes and a few studies with statins, but there is no comprehensive proteomic study was reported with combined treatment of LPS and statin on macrophage cells. Large-scale studies in functional genomics or proteomics produce a huge amount of data that are difficult to analyze by conventional methods^32^. Analysis of biological networks using differentially expressed proteins after the treatment of statin and LPS in Raw 264.7 macrophage cells can predict possible canonical pathways, upstream regulators, and functional metabolic networks. Analyses of large-scale data for a system is helping to discover several protein targets for further functional validation.

This study presents a discovery based label-free quantitative proteomics due to the combined treatment of LPS and statin on murine Raw 264.7 macrophage cells. In this study, Raw 264.7 macrophage cells were stimulated by the proinflammatory mediator LPS as well as drug statin to understand the regulation and mechanism of proteins comprehensively during inflammatory response, and the relative effect of LPS and statin on the protein level. This study included an effective compilation of statin and LPS treated liquid chromatography-mass spectrometry (LC-MS/MS) proteomics data and elucidates their correlation with the anti and proinflammatory responses of immune cells. We also narrowed down a significant protein target, plectin by proteomics study and further validated its expression by biochemical and molecular biological methods and provided convincing evidence of its influence due to statin drug and pathogenic stimuli.

## Results

### Identification and quantification of LPS and statin modulated proteins

**Figure 1:**
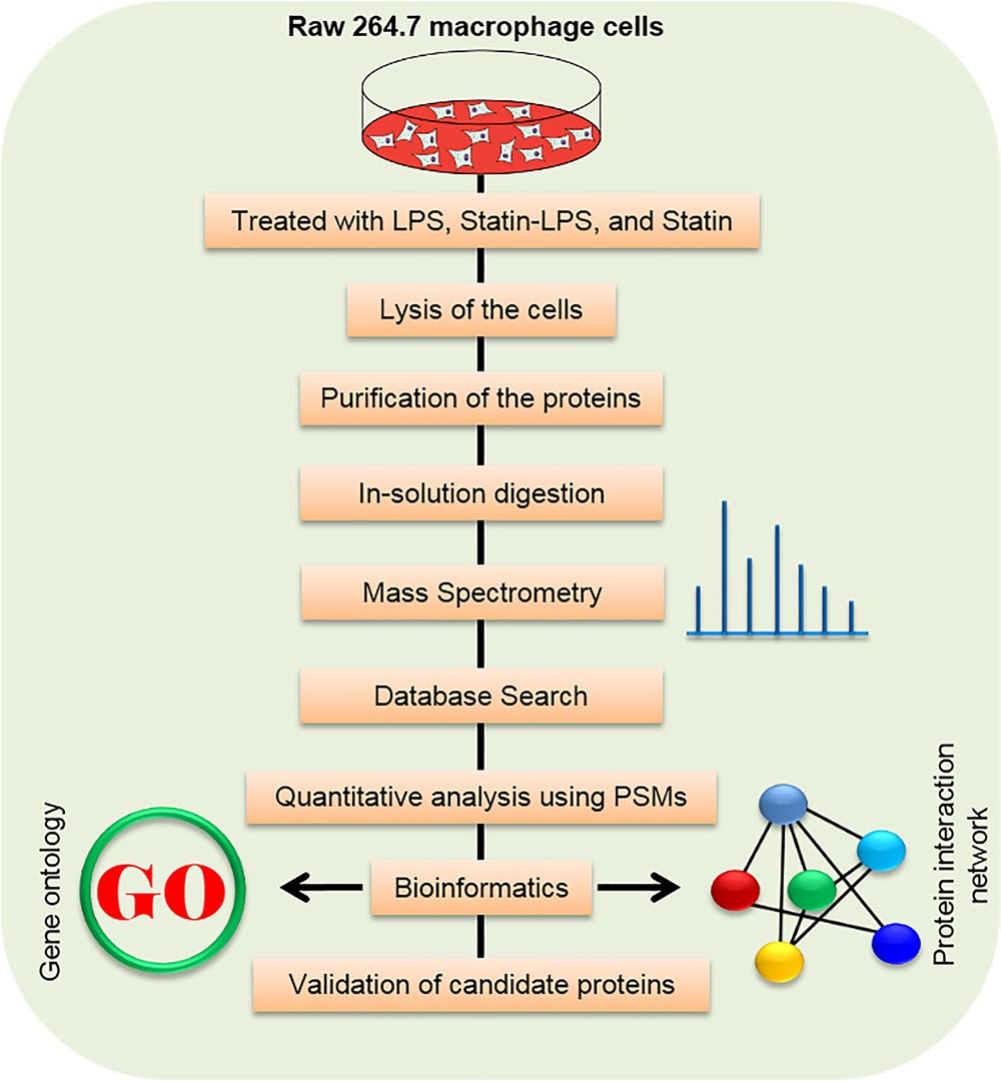
Schematic diagram of proteomic analysis of Raw 264.7 macrophage cells with the treatment of LPS and statin.

To investigate the comprehensive effect of LPS and drug statin in macrophages, we utilized a gel-free quantitative proteomic approach with high-throughput mass spectrometry in Raw 264.7 macrophage cells. A complete scheme of the study is shown in Fig. 1. Four treatments were employed as: control, LPS, statin-LPS, and statin, respectively. Total proteins were extracted from the treated cells, then digested using Trypsin and were analyzed by nano-LC-MS/MS. Three biological replicates were performed for this proteomics study. The raw LC-MS/MS spectra were searched for identifying the proteins from UniProt mouse protein sequences. After searching the spectra with proteome discoverer software, peptides were quantified using peptide-spectrum matches (PSMs). PSMs are the total number of identified peptide sequences for a protein including those redundantly identified. Figure S1 shows the comparisons of correlation matrix and their pairwise correlation coefficient among three biological replicates in each sample. Here, significant correlation coefficients are shown with a value of *R* ^2^ ≥ 0.80 among the biological replicates.

**Figure 2:**
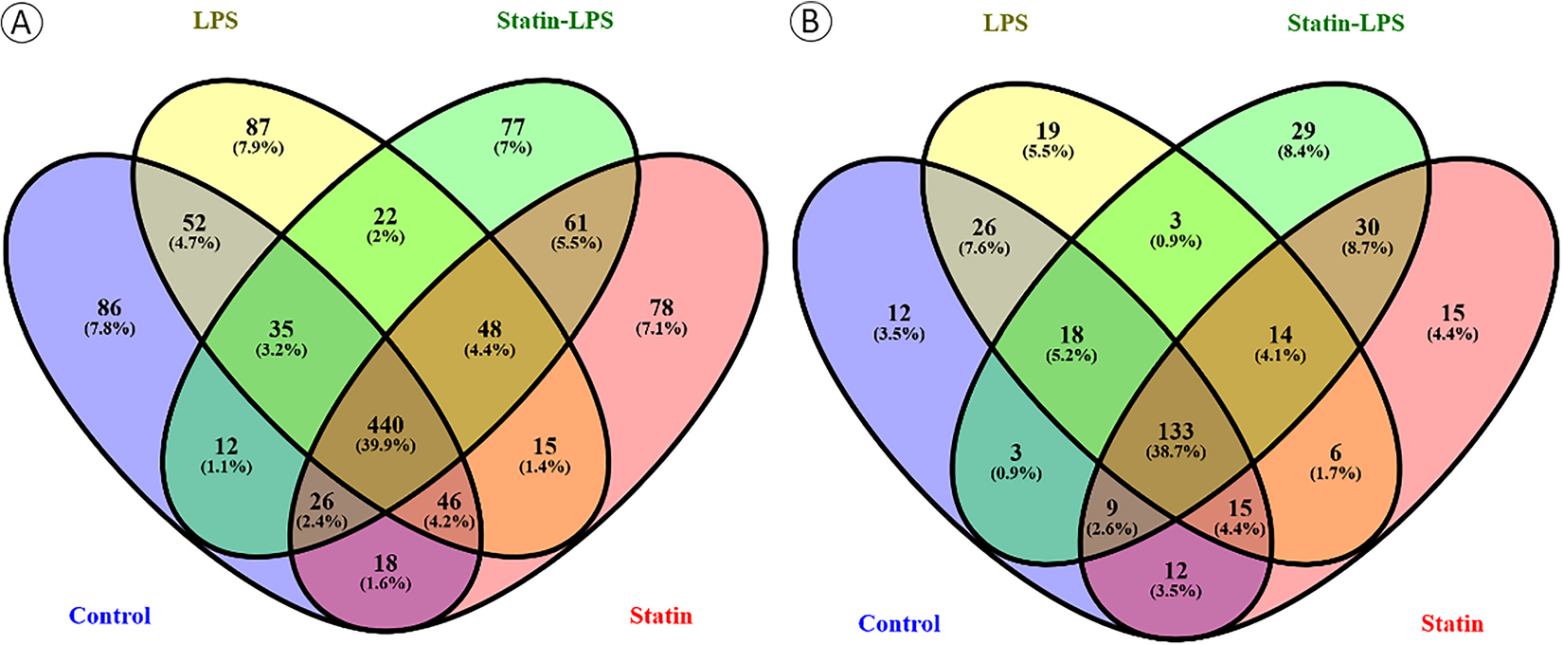
Venn diagram of identified proteins with one or more than one peptides (**A**) and two or more than two peptides (**B**) per proteins in the treatment of LPS and statin in macrophage cells.

A total number of 1103 proteins was identified with ≥1 peptide match without filtering the LC-MS/MS proteomics data sets in Raw 264.7 macrophage cells. Among these 1103 proteins, a total of 715, 745, 732, and 721 proteins were identified in control, LPS, statin, and statin-LPS treated samples in macrophage cells, whereas 440 proteins were commonly identified in all the treatments (Fig. 2A). After strict filtering of the data sets, we decided to keep a subset of 344 proteins whose proteins expression were quantified. The filtered proteins were presented in three biological replicate samples with at least 2 peptides/proteins using high confidence search criteria (please see the data analysis section) and a false discovery rate of 1%. Detailed information about the identification of proteins and peptides is shown in Tables S1 and S2. Out of these 334 proteins, 133 proteins were commonly shared among control, LPS, Statin-LPS, and statin treatments, and 136 proteins were shared in any of three treatments, whereas a total of 75 proteins were exclusively identified (12 proteins in control, 19 proteins in LPS, 29 proteins in statin-LPS, and 15 proteins in statin) in each treatment in Raw 264.7 macrophage cells (Fig. 2B).

**Figure 3:**
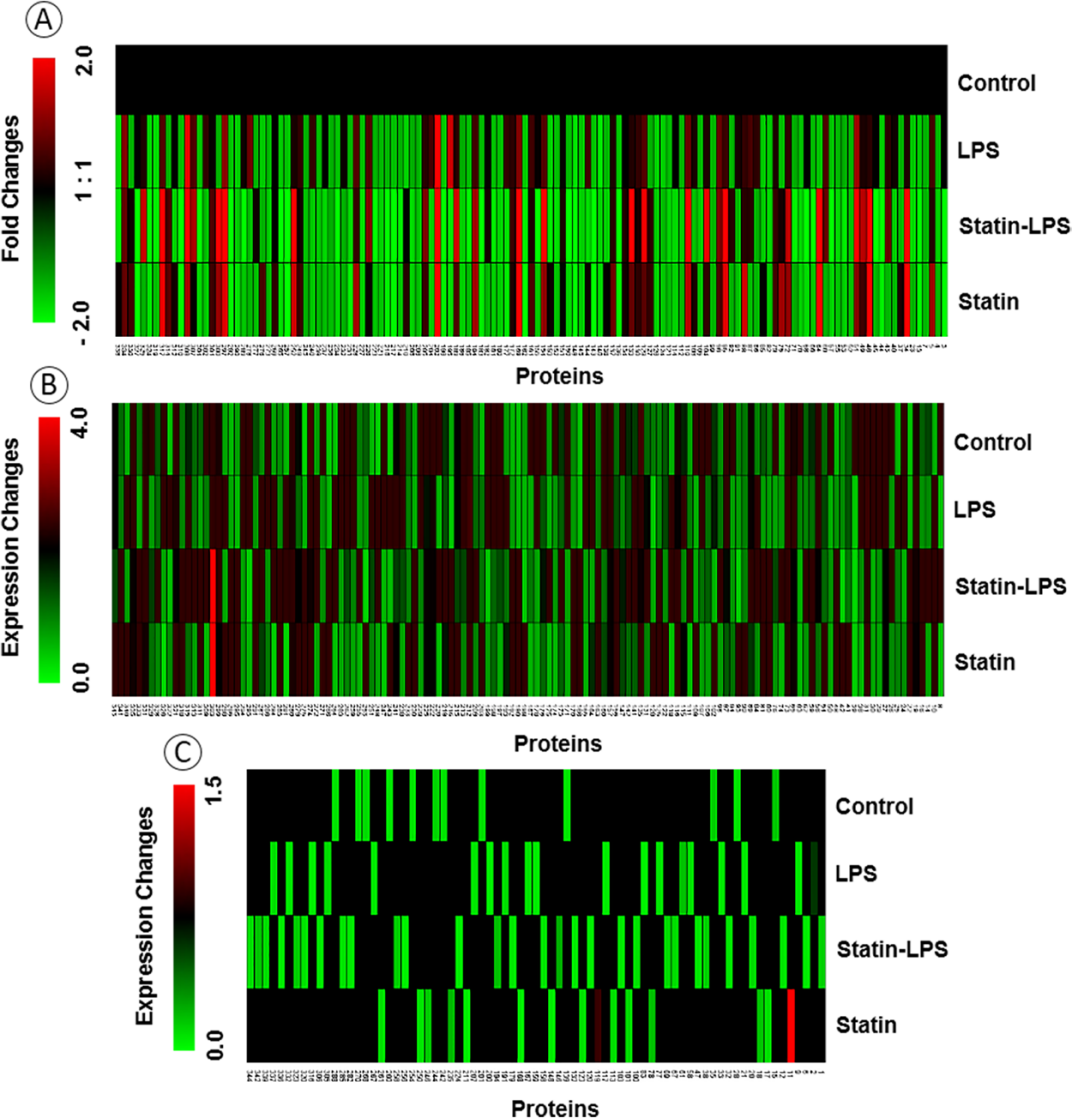
Heat map of differentially expressed proteins at the treatment with LPS and statin in Raw 264.7 macrophage cells. The heat map was generated using MeV software with the normalized intensities of highly confident peptide spectra matches (PSMs) in proteins. Differences in the intensity of proteins commonly shared among control, LPS, Statin-LPS, and statin (**A**), shared among any of three treatments (**B**), and exclusively identified proteins are presented in clusters to form (**C**). The numbers indicated the protein information’s in Tables S1 and S2.

The differential protein expressions were displayed by normalized PSMs as a heat map. Their abundances are considered for protein quantitation during the mass spectrometry analysis of complex samples. One-hundred-thirty-three proteins identified in all treatments that are illustrated by the heat map based on their protein abundances (Fig. 3A), whereas 136 protein intensities showed in any of three treatments (Fig. 3B). Also, a total of 75 proteins, which were expressed exclusively in four treatments is showed in Fig. 3C. Those heat maps provided us with a clear portrait of the protein expression changes during the treatment of statin and LPS in Raw 264.7 macrophage cells.

**Figure 4:**
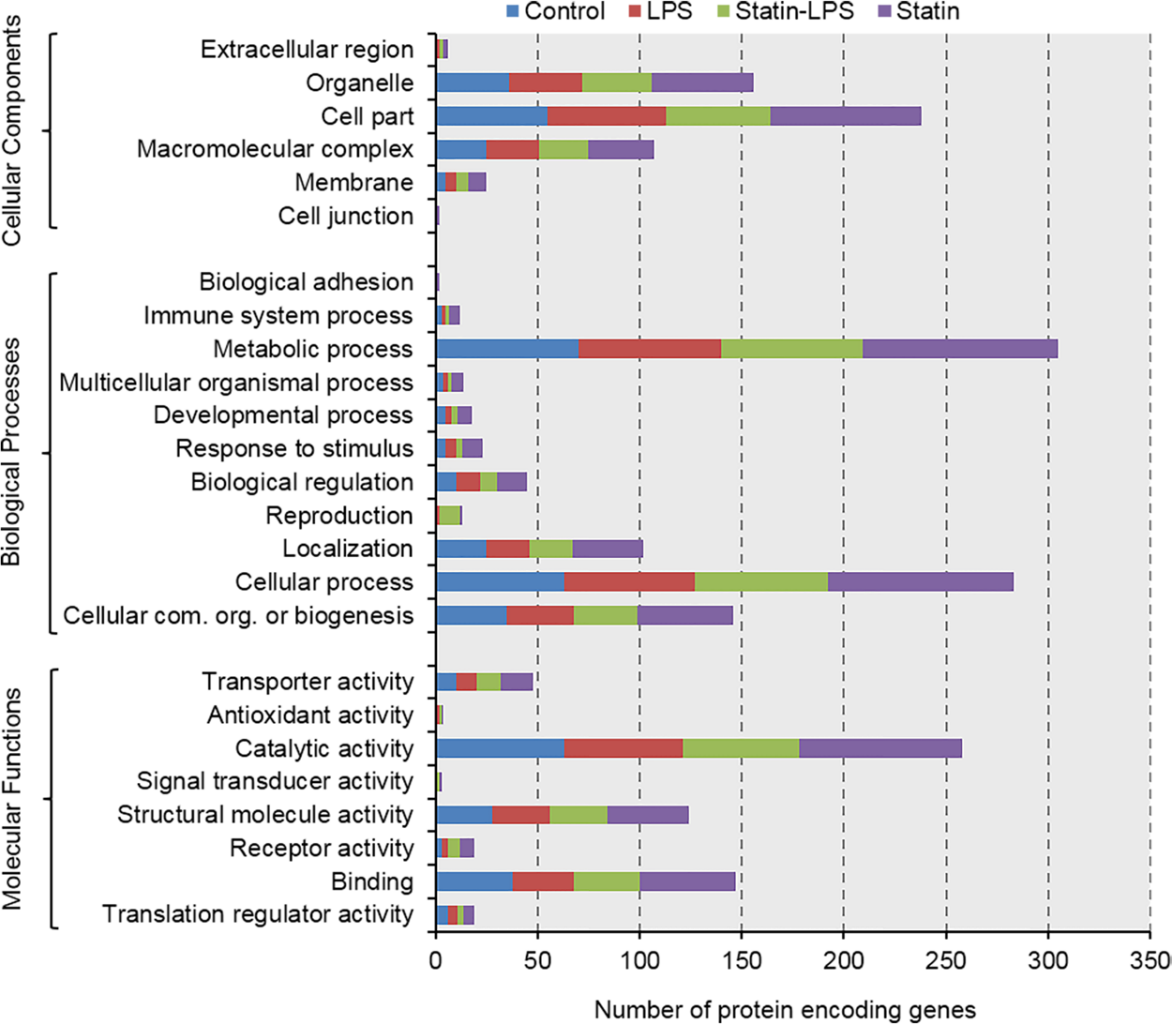
Overview of functions of protein-encoding genes. Analysis of gene ontology based on biological processes, molecular functions, and cellular components using the PANTHER classification system.

All filtered proteins were evaluated for gene ontology classifications, such as molecular functions, cellular component, and biological process using PANTHER gene classification systems^33^. According to the “molecular function” most of the proteins belong to the categories of catalytic activity, protein binding, and structural molecule activity. According to the “biological process” most of the proteins belong to the categories of metabolic process, cellular process, and cellular component organization or biogenesis. For “cellular component” classification, most of them were localized in cell part, macromolecular complex, and organelle (Fig. 4).

### Plectin- and prohibitin 2- based targeted network

**Figure 5:**
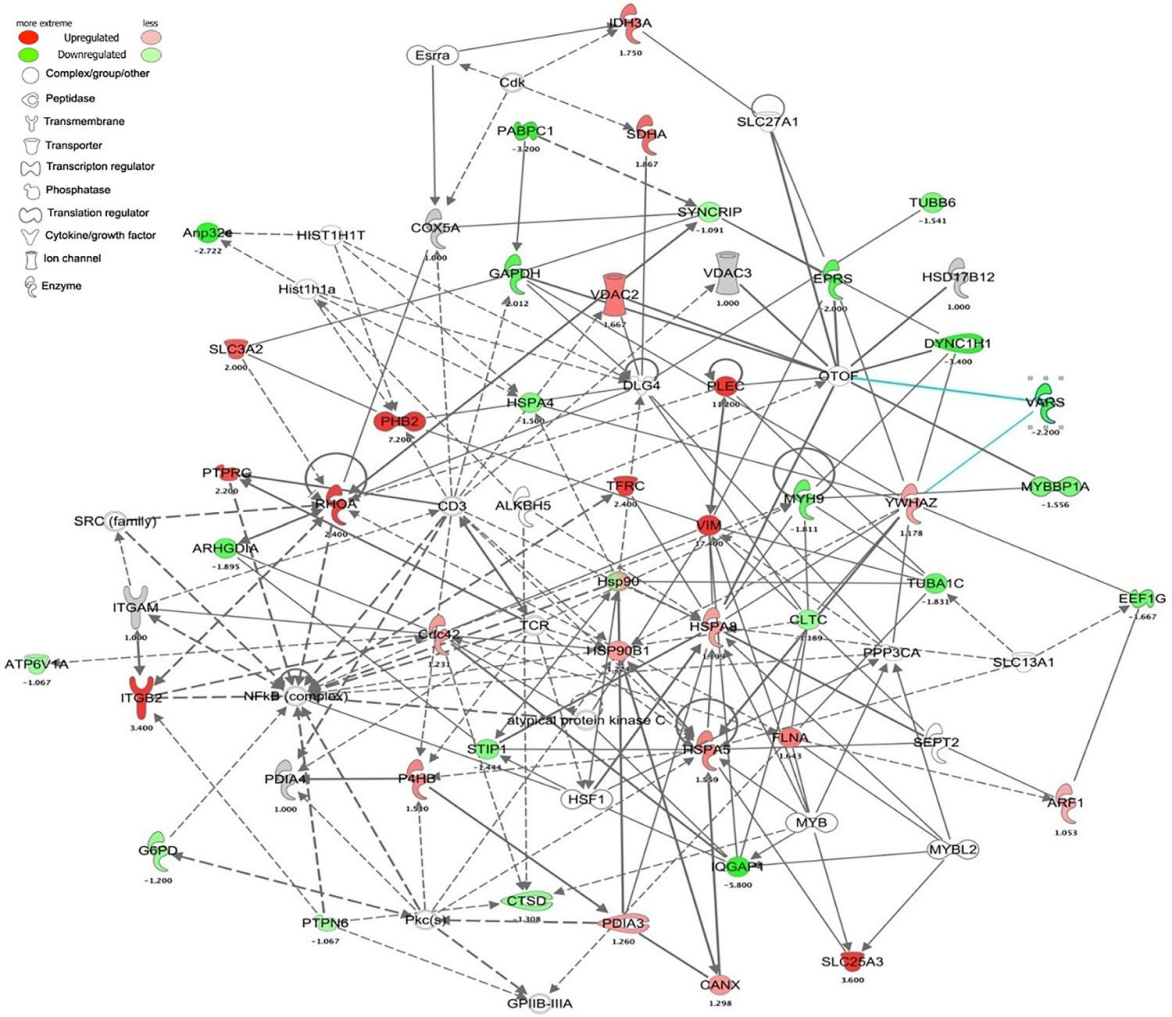
Plectin- and prohibitin 2-based targeted protein network with the stimulation of LPS and treatment of statin in Raw 264.7 macrophage cells. The networks were generated using Ingenuity Pathways Analysis (IPA) bioinformatics software in mouse databases from the statin treatment. Direct and indirect interaction are represented by the solid, and dashed line, respectively. The shapes represent the molecular classes of the proteins defined in the legend. The proteins interactions networks were generated through the use of IPA (QIAGEN Inc., https://www.qiagenbio-informatics.com/products/ingenuity-pathway-analysis)^34^.

Core analysis of protein was implemented by Ingenuity® Pathways Analysis (IPA, Ingenuity Systems, www.ingenuity.com), where proteins are analyzed as a network using canonical pathways, involvement in disease networks and predicted upstream regulators. Interconnecting proteins were displayed as a protein network, namely protein-to-protein interactions^34^. From IPA analysis of our experimental proteome datasets, 24 protein networks were predicted. Plectin and prohibitin 2 proteins were ranked second and fourth in that analysis and the ranking was generated based on number of focused molecules identified in that networks. Due to their high expression in treatments compared to control, proteins plectin and prohibitin 2 were selected for targeted interactome analysis. Plectin (Plec)- and prohibitin 2 (PHB 2)-based network were generated by IPA considering the fold changes of proteins using a mouse database (Fig. 5). Here, we found Plec is directly interacting with vimentin, tubulin alpha 1 C, and otoferlin (OTOF), while indirectly interacting with Ras homolog (Rho) gene family member A (Fig. 5). Compared to other proteins, Plec is found highly abundant in the treatment of statin; however, vimentin expression was increased with LPS treatment but decreased in statin treated macrophages. The tubulin alpha 1C chain protein was down-regulated by the treatment of LPS and statin, while the RhoA protein was only expressed in statin-LPS and statin-treated samples. PHB 2 was found directly interacting with vimentin, heat shock protein A4 (HSPA4), and CD98 heavy chain proteins (CD98); however, PHB 2 indirectly interacted with histone H1.1 (HIST1H1A) and H1t (HIST1H1T), and CD3 protein (CD3). HSPA4 were interacted indirectly with heat shock protein 90 (HSP90). Compared to LPS and statin alone, PHB 2 expression was highly increased in the statin-LPS treated sample; however, compared to the control, its expression was high in LPS and statin. HSPA4 was down-regulated in the treatment of LPS and statin alone in Raw 264.7 macrophage cells (Figs 5 and S2, Tables S1C and S2C).

### Inflammatory disease based network

**Figure 6:**
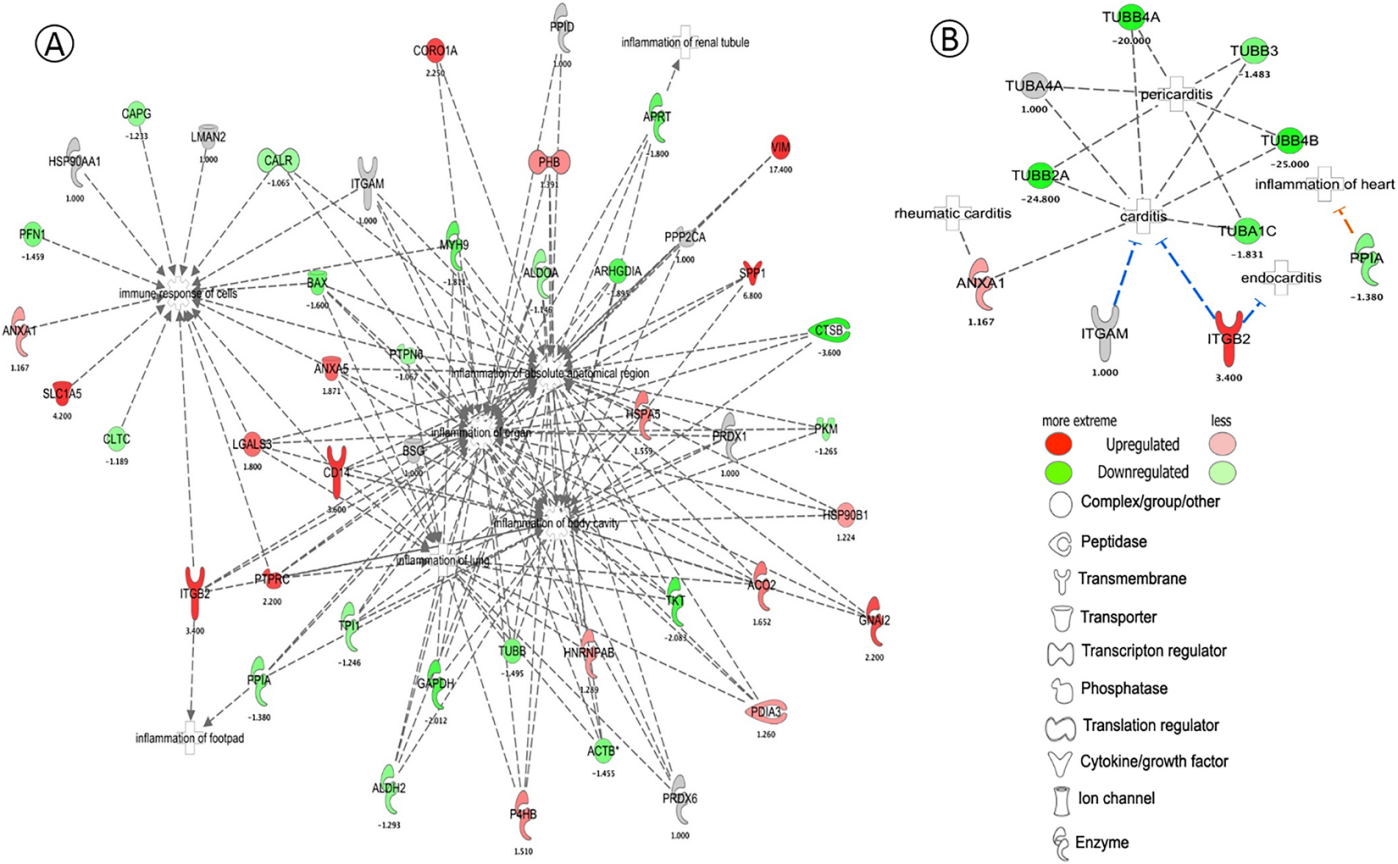
IPA-based protein network involved in inflammatory diseases based on inflammation responses (**A**) and cardiac inflammations (**B**) in Raw 264.7 macrophage cells, which are treated with statin and stimulated by LPS. Network was generated by IPA using the statin treated data. The shapes represent the molecular classes of the proteins as defined in the legend. The functional networks of proteins were generated through the use of IPA (QIAGEN Inc., https://www.qiagenbio-informatics.com/products/ingenuity-pathway-analysis)^34^.

A disease and function-based protein network was generated with the identified proteins (Fig. 6). This network pointed out relationships of several inflammatory diseases and their functions to Raw 264.7 macrophage cells in the treatment of LPS and statin, such as immune response of cells, inflammation of renal tubule, inflammation of absolute anatomical region, inflammation of organ, body cavity, lung, and footpad. A putative uncharacterized protein (Hsp90aa1) was categorized in the immune-response functional network of cells, which was highly expressed in LPS and statin-LPS, but unchanged in statin treated samples. Monocyte differentiation antigen (CD14), galectin-3 (LGALS3), annexin A5 (ANXA5), and osteopontin (SPP1) were the part of the interaction with inflammation of organs, lungs, body cavities, and absolute anatomical regions. CD14 and SPP1 were increased in statin-LPS and statin, but did not change in the LPS treatment. LGALS3 and ANXA5 expression were found increased in all three treatments. Coronin (CORO1A) and prohibitin (PHB) were the part of the interacting network of inflammation of organs and absolute anatomical regions. PHB expression was increased during the stimulation of LPS, statin-LPS and statin but CORO1A was decreased during LPS treatment. Vimentin, an interacting partner of Plec, is related to inflammation of absolute anatomical regions and organs (Fig. 6A, Tables S1 and S2). Some identified proteins in the treatment of LPS and statin were associated with cardiac inflammation (Fig. 6B, Tables S1 and S2). Tubulin family proteins, namely subunits of B4A, A4A, B3, 2A, 4B, and A1C were involved with pericarditis and carditis. Here, anexin A1 is related to carditis and rheumatic carditis, whereas integrin beta-2 (ITGB2) has been reported as being able to suppress carditis and endocarditis^35^. Tubulin B4A, B3, 2A, 4B, and A1C were down-regulated after treatment of LPS and statin, but tubulin A4A was highly up-regulated during the stimulation of LPS in Raw 264.7 macrophage cells. Interestingly, ITGB2 was up-regulated as the treatment of statin, but did not change when stimulated by LPS. Peptidyl-prolyl cis-trans isomerase A (PPIA) was found down-regulated in the treatment of LPS and statin in macrophage cells, which function was related to the inflammation of heart based on IPA prediction (Fig. 6B, Tables S1 and S2).

### Targeted-proteins validation by immunoblotting and immunocytochemistry

**Figure 7:**
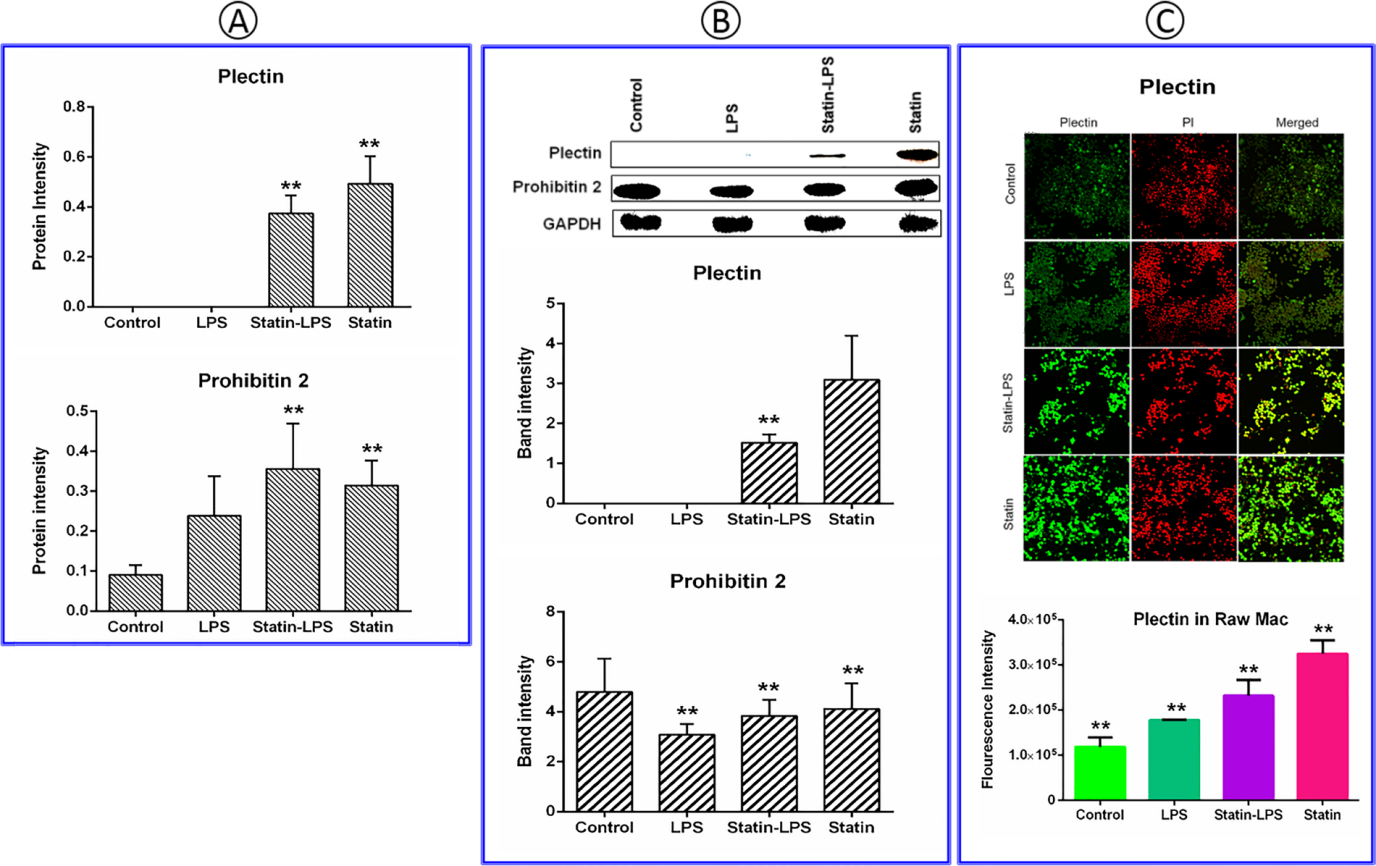
The mass spectrometry data (**A**) of candidate proteins, plectin and prohibitin 2, which were validated in the treatment of LPS and statin in Raw 264.7 macrophage cells. Immunoblotting and densitometric analysis were performed using ImageJ for quantifying the band intensity (**B**). Plectin was further validated using immunocytochemistry, and intensities were calculated by ZEN lite software in the bar graph. Here, Propium iodide (PI) was used as a control (**C**). GAPDH was used as loading control. All data are presented as mean ± SEM (n = 3 in each group) with *P < 0.05, as per student’s t-test.

According to the proteomic data, a large number of proteins differentially expressed in Raw 264.7 macrophage cells due to the treatment of LPS and statin. Two highly modulated proteins plectin and prohibitin 2, were selected for further validation by immunoblotting. Plectin protein was highly expressed in the samples treated by statin-LPS and statin, but did not express in control and LPS. However, prohibitin 2 was highly expressed in the statin-LPS treated sample (Tables S1A and S2A). Due to its high expression in the statin treated samples, plectin protein was also cross-validated using immunocytochemistry with selected antibody followed by confocal microscopy. Expression levels detected by immunoblotting for plectin and prohibitin 2 proteins were found highly correlated with the mass spectrometry data (Figs 7A, B and S3). Moreover, plectin protein expression was visualized by confocal microscopy, which was found similar to the results of mass spectrometry and immunoblotting (Figs 7A–C and S3).

**Figure 8:**
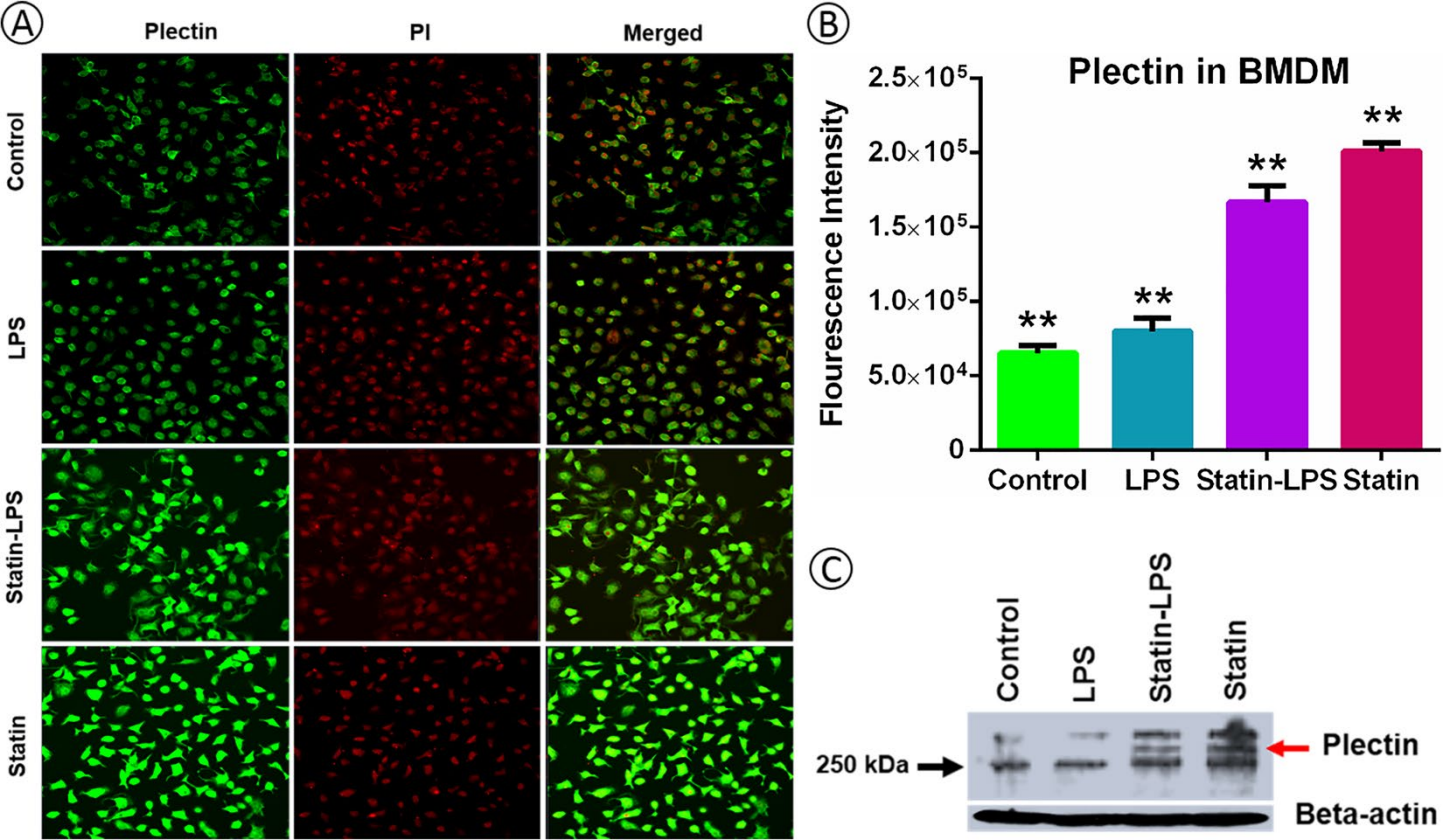
Validation of plectin protein expression on the BMDM cells upon the treatment of LPS and statin. Immunocytochemistry analysis of BMDMs stained with anti-plectin (**A**), where fluorescence intensities were quantified using ImageJ (**B**). Here, propium iodide (PI) was used as control for immunocytochemistry. Moreover, plectin protein was further validated using immunoblotting (**C**). Beta-actin was used as a loading control. All data are presented as mean ± SEM (n = 3 in each group) with **P < 0.05, as per student’s t-test.

In order to validate the plectin protein expression in primary macrophages, we cultured the mouse bone marrow derived macrophages (BMDMs) upon the treatment of LPS and statin. We further verified the plectin protein expressions by immunocytochemistry and immunoblotting (Figs 8A–C and S4). Plectin is a large protein, so immunoblotting data was further verified with immunocytochemistry. Plectin protein expressions in BMDMs were quantified by confocal microscopy comparing the fluorescently labeled plectin protein in each treatment (Fig. 8B). The expressions of plectin protein by immunocytochemistry and immunoblotting were found very similar in both raw and primary BMDM macrophages (Figs 7, 8, S3, and S4).

### F-actin cytoskeleton response to statin by plectin knockdown in Raw 264.7 macrophages

**Figure 9:**
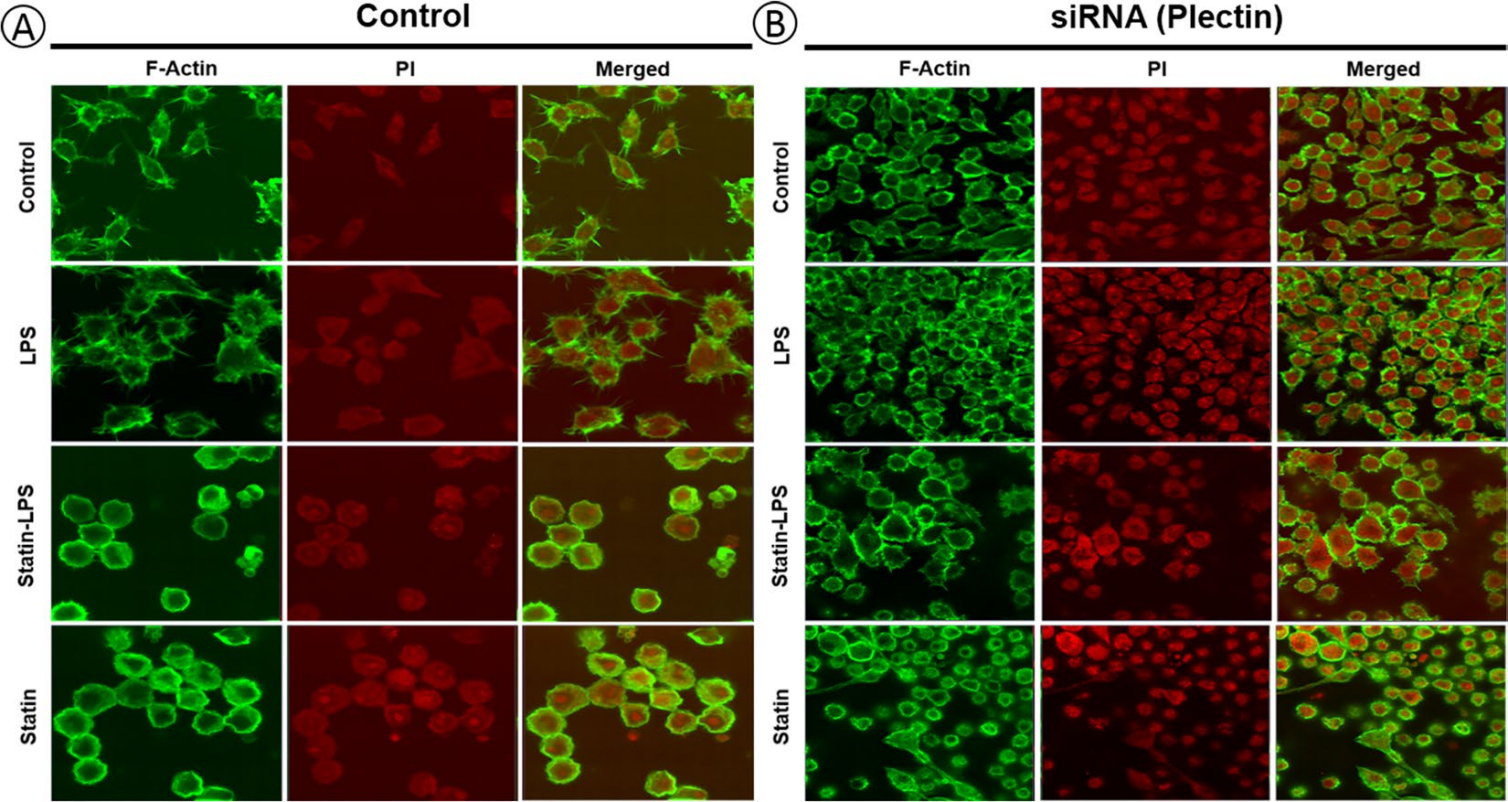
Plectin protein influenced the filamentous-actin (F-actin) rearrangement upon the treatment of LPS and statin. Phalloidin labeled F-actin cytoskeleton was studied by immunocytochemistry using Raw 264.7 macrophage cells (control) (**A**), and plectin knockdown Raw 264.7 macrophage cells (**B**). Propium iodide (PI) was used for nuclear staining.

To understand the functions of plectin protein upon the treatment of LPS and statin in Raw 264.7 macrophage cells, we used knockdown plectin by siRNA. To examine how statin drugs affect the cell morphology and filamentous-actin cytoskeleton, we designed an imaging study with confocal microscopy. F-actin cytoskeleton was stained by phalloidin-alexa-flour 488 in plectin knockdown and non-knockdown Raw 264.7 macrophage cells (Fig. 9). From the cell morphology image, it is clear that statin drug impacted F-actin cytoskeleton compared to the control and LPS-induced macrophage cells (Fig. 9A). F-actin cytoskeleton was also deformed in plectin inhibited macrophages but cell shape was further affected by statin-treated cells (Fig. 9B). It is clear that plectin protein plays an important role in organizing the F-actin cytoskeleton in macrophages and that statin drug significantly affects cytoskeletal networks.

## Discussion

In this study, large-scale proteomics investigations have been emphasized to identify functions of protein encoding genes and protein-protein interaction networks and accomplishing a more inclusive understanding of the cell’s response to a wide range of stimuli. Additionally, these studies generally open a new avenue to discover novel targets for therapeutics. Particularly, protein expression analysis is very important among the large-scale approaches, because the restricted messenger RNA identification using microarray analysis does not always correlate with the protein expression^36^. In this study, we quantified 344 proteins using label-free MS-based proteomics in murine Raw 264.7 macrophage cells after the treatment of a microbial ligand LPS and the drug statin (Fig. 2B, Tables S1 and S2). After cell lysis, a methanol-chloroform protein precipitation was performed to remove the detergent from the cell lysate. Stringent filtering was done to provide high confidence in datasets, which further reduced the number of proteins ID in our LC-MS/MS data. Protein quantitation were performed utilizing the number of PSMs observed for per protein due to the treatments. Protein abundances as well as exclusively identified proteins in different treatments were visualized with heatmaps. (Fig. 3). According to gene ontology classification, we did not find any significant changes among the treatments in Raw 264.7 macrophage cells. Majority of the identified proteins based on protein encoding gene ontology were highly influenced by the treatment of statin in Raw 264.7 macrophage cells (Fig. 4).

Our proteomics data set has also been analyzed to unveil potential changes at protein level in disease conditions, especially inflammation-related molecular functions. Statin treatment increased the expression of annexin A5 (ANXA5), integrin beta-2 (ITGB2), and osteopontin (SPP1) proteins, and our IPA analysis predicted their association with the inflammatory functions in the cells (Fig. 6A and B). Annexin A5 is a protein from the annexin family and generally expresses in extracellular and intracellular locations. Annexin A5 has high affinity to membranes bearing phosphatidylserine (PS)^37^. PS prompts phagocytosis through stimulating PS receptors precisely on the macrophage or by binding between molecules like annexins A1 and A2^38^. ANXA5 does not act as a uniting molecule, such as annexins A1 and A2. ANXA5 inhibits the phagocytosis of dying cells that require a high concentration of ANXA5^39^. ANXA5 performs a function *in vivo* in the inflection of the immune system by hindering phagocytosis of apoptotic and necrotic cells^40^. Thus, extracellular ANXA5 may play an important role in activating the macrophages to produce inflammatory factors during the treatment of LPS and statin interactions for therapeutic application.

ITGB2 has also been known as leukocyte function-associated antigen-1 (LFA1) and is associated with leukocyte adhesion and transmigration including T-cells and neutrophils^41^. In this study, ITGB2 was increased upon the treatment of statin-LPS and statin, but did not changes in LPS-stimulation in macrophage cells (Fig. 6B). ITGB2 has also acted as an adhesion molecule and initiator of intracellular signaling pathways that contributes to phagocytosis, especially in the removal of apoptotic cells^42^. Statin drugs regularly used for the management of hypercholesterolemia specifically by blocking LFA-1-mediated adhesion and co-stimulation of lymphocytes which inhibits the inflammatory response through active LFA-1 inhibitors^43^. Finally, ITGB2 might be a potential candidate in an autoimmune disease that might be contributing to the inflammatory responses.

A very important observation of our study is the high expression of plectin due to the treatment of statin. Plectin is versatile cytoskeletal linker proteins of multi-domain and enormous size (534 kDa) and highly expresses in a broad range of cell types and mammalian tissues^44^. Plectin not only play a key role in maintaining tissues, cell integrity, coordinating dynamic alterations in cell shape and cytoarchitecture but also provides protein scaffolding platforms for the positioning, assembly, and direction of signaling complexes^45,46^. They have proven essential to the integrity of muscle cells, including connection between the intermediate filaments to hemi-desmosomes and desmosomes in epithelial cells^47^ and to crosslinking of actin filaments and microtubules in nervous systems^45^. Plectin relate to vimentin from the initial stages of filament assembly by serving as a crosslinker of vimentin filaments^48^. They are necessary for the development of intermediate filaments and their directional movement towards the periphery of cells^49^. Plectin is anchoring and stabilizing the molecular process for cancer cell invasion and extravasation for metastasis by vimentin intermediate filament (IF) scaffolds^50^. The function of cyto-linker plectin protein was to join the IF networks to a nuclear envelope and to cytoplasmic organelles that mediate their crosstalk between actin and tubulin cytoskeletons. Due to these important roles, plectin significantly influences the formation and dynamics of IF networks and their cytoarchitecture^51^. Simvastatin caused a rapid and profound perinuclear bundling of vimentin and reduced the area of the vimentin IF network relative to vehicle treatment without significant changes in vimentin protein solubility in invasive cancer cells^50,52,53^. These findings are significantly consistent with our results in Raw 264.7 macrophage cells during the treatment of LPS and statin. The impact of statin among plectin, vimentin, and tubulin proteins were visualized by a network (Fig. 5). Notably, vimentin was a LPS-responsive protein, which was not significantly changed in statin treatment in macrophage cells compared to LPS and statin-LPS treatment (Figs 5 and S2). Plectin also has a secondary as well as an indirect relation with integrin beta 2 (β2) via Rho A protein, which was highly expressed at the treatment of statin in Raw 264.7 macrophage cells (Fig. 5). Increased integrin β2 may correspond to an essential element of the vascular-protective and anti-inflammatory substances of statins and serve as a novel approach to maximize inflammatory vascular syndromes^14^. Our study clearly showed direct relation among plectin, vimentin, and tubulin proteins due to the treatment of statin in murine Raw 264.7 macrophage cells (Figs 5, and S2), whereas all three interacting partners has been associated to cytoskeleton network, microtubules, and IF assembly. Plectin was highly expressed in statin treated cells; however, they were not changed during the treatment by LPS in Raw 264.7 macrophage and BMDM cells (Figs 7B,C, 8A–C, S3 and S4). The data evidently showed statin and LPS have an antagonistic effect on the plectin protein during inflammation (Fig. 7B). To study the plectin function, we used knockdown plectin in raw macrophage cells. We found that filamentous actin cytoskeleton was significantly impacted by statin treatment and cell morphology was changed significantly. In the knockdown cells, we noted that the cell shape was also changed after plectin knockdown and was more disorganized after statin treatments. It is clear that statin has a significant impact on the cytoskeletal network of the cells. The main function of plectin is stabilizing and linking intermediate filament networks. From these experimental data, it is clear that plectin might have a novel influence on assembling and mobilizing the intermediate filaments. A complete functional study on plectin is anticipated from this outcome in near future (Fig. 9).

## Conclusions

A gel-free quantitative proteomic profiling in murine Raw 264.7 macrophage cells along with the treatment of statin (simvastatin) and LPS-stimulation allowed to understand the changes of protein expression and proteins activities. We identified 334 differentially expressed proteins and 133 commonly shared proteins with high confidence in LPS and statin treated macrophages. Their subsequent analysis by bioinformatics tools provided us to understand the biological, molecular, and functional activities during the combined treatment of statin-LPS in macrophage cells. Functional protein networks and disease based networks were provided insight about the effect of statin and LPS on immune cell macrophage. Protein interaction networks exhibited the relationship among plectin, vimentin, and tubulin proteins, which provided the impact of cytoskeletal proteins due to the combined or individual treatments. Moreover, inflammatory disease based protein networks unveiled several proteins, (for example ITGB2, SPP1, and ANXA5) and their impact in the regulation of inflammatory diseases. Mass spectrometry-based proteomics data of highly expressed plectin protein were further validated by western blotting and immunocytochemistry, which provided convincing data of the impact on this protein due to the statin treatment. Plectin effects on F-actin cytoskeletal networks were studied using knockdown and further imaging studies. Finally, in this study, the identified proteins and their expressions provided a basis for better understanding of the functional and regulatory importance of inflammatory mechanisms as well as the combined effect of statin and LPS on inflammation at protein levels. Due to high expression of plectin in statin treated macrophages, we recommend that future studies are required to uncover the plectin’s role in statin mediated inflammation. We anticipate submitting a new manuscript on plectin functions in near future.

### Experimental procedures

Raw 264.7 macrophage cells were a gift from Dr. Michael B. Fessler, Immunity, Inflammation and Disease Laboratory, NIEHS, NIH, Research Triangle Park, North Carolina, United States. DMEM medium was purchased from CORNING, VA, USA. Trypsin (V5111) was obtained from Promega, WL, USA. Lipopolysaccharide (tlrl-3pelps) was from InvivoGen, California, USA. We bought Simvastatin (S6196) from SIGMA-ALDRICH, MO, USA. Plectin (ab83497 and ab32528), goat anti-rabbit Alexa Flour 488 (ab15007), and phalloidin-iFluor 488 (ab176753) were purchased from Abcam, MA, USA. Anti-prohibitin 2 (SC-67045) was from Santa Cruz Biotechnology, Dallas, TX and transfection reagent of siTRAN (TT300001) was from OriGene, MD, USA.

#### Mice

Wild-type (C57BL6/J) mice were purchased from Jackson Laboratory and maintained in a specific pathogen free (SPF) facility at UT Southwestern (UTSW) Medical center. Collection of bone marrow macrophages cells protocol was approved by the UTSW medical center Institutional Animal Care and Use Committee (IACUC) and were conducted in accordance with the IACUC guidelines and the National Institutes of Health Guide for the Care and Use of Laboratory Animals.

### Cell culture and treatments

The murine RAW 264.7 macrophage cells were cultured and maintained in Dulbecco’s Modified Essential Medium (DMEM). Cells were grown at 37 °C in DMEM medium supplemented with 10% fetal bovine serum, 1% penicillin/streptomycin in a humidified atmosphere of 5% CO_2_.

For culturing of bone marrow derived macrophages (BMDM), bone marrows collected from mouse femurs and tibias were cultured in L-cell-conditioned IMDM medium supplemented with 10% FBS, 1% nonessential amino acid, and 1% penicillin-streptomycin for 6 days to differentiate into macrophages^54^. BMDMs were sub-cultured in 6-well plate for immunocytochemistry as well as immunoblotting. Cells were seeded in culture plate and the medium was replaced at every 2–3 days. Cells were treated with simvastatin (Sigma) at 10 µM of final concentration for 24 hr, then stimulated with LPS at 1 µg/ml (tlrl-3pelps, InvivoGen) in fresh medium, and incubated at 37 °C for 1 hr. Cells were rinsed with phosphate buffer saline (PBS) three times, then scraped for collection.

### Protein preparation, purification, and digestion

The cells were incubated in RIPA buffer with protease inhibitor at 4 °C for 15 mins, then sonicated for another 15 mins. The suspended cells were placed at 4 °C for 30 mins, then centrifuged at 20000 × g for 30 mins at 4 °C. The supernatant was collected into a new tube; then, the protein concentration was measured with BCA protein assay using bovine serum albumin standard.

The extracted proteins (150 μg) from Raw 264.7 macrophage cells were purified using a methanol-chloroform method according to Kamal *et al*.^21^. The dried pellet was resuspended in 50 mM NH_4_CO_3_. According to Chakrabarty *et al*.^55^, proteins were reduced and alkylated, then digested with Trypsin (MS Grade) at a 1:50 enzyme/protein concentration for 16 h at 37 °C. Formic acid (pH < 3) was added to the resulting peptides for acidifying the sample. A C_18_ desalting column (ThermoFisher Scientific, IL, USA) was used for desalting the samples. After completely drying by speed vacuum, peptides were dissolved in 0.1% formic acid, and stored at −20 °C.

### Mass analysis (nano-LC-MS/MS)

Digested peptides were analyzed by nano-LC-MS/MS using a Velos Pro Dual-Pressure Linear Ion Trap Mass Spectrometer (ThermoFisher Scientific, MA) coupled to an UltiMate 3000 ultra-high-performance liquid chromatography (UHPLC, ThermoFisher Scientific, MA). Peptides were loaded onto the analytical column and separated by reversed-phase chromatography using a 15-cm column (Acclaim PepMap RSLC) with an inner diameter of 75 μm and packed with 2 μm C_18_ particles (Thermo Fisher Scientific, MA). The peptide samples were eluted from the Nano column with multi-step gradients of 4–90% solvent B (A: 0.1% formic acid in water; B: 95% acetonitrile and 0.1% formic acid in water) over 70 min with a flow rate of 300 nL/min with a total run time of 90 min. The mass spectrometer was operated in positive ionization mode with nano spray voltage set at 2.50 kV and source temperature at 275 °C. The three precursor ions with the most intense signal in a full MS scan were consecutively isolated and fragmented to acquire their corresponding MS2 scans. Full MS scans were performed with 1 micro scan at resolution of 3000, and a mass range of m/z 350–1500. Normalized collision energy (NCE) was set at 35%. Fragment ion spectra produced via high-energy collision-induced dissociation (CID) was acquired in the Linear Ion Trap with a resolution of 0.05 FWHM (full-width half maximum) with an Ultra Zoom-Scan between *m/z* 50–2000. A maximum injection volume of 5 µl was used during data acquisition with partial injection mode. The mass spectrometer was controlled in a data-dependent mode that toggled automatically between MS and MS/MS acquisition. MS/MS data acquisition and processing were performed by Xcalibur^TM^ software (ThermoFisher Scientific, MA).

### Data analysis

Proteins were identified through Proteome Discoverer software (ver. 2.0, ThermoFisher Scientific) using UniProt mouse (*Mus musculus*) protein sequence database (75568 sequences, and 32232886 residues). The reviewed protein sequences of mouse were downloaded from UniProt protein database (www.uniprot.org) at January 15, 2016. The considerations in SEQUEST searches for normal peptides were used with carbamidomethylation of cysteine as the static modification and oxidation of methionine as the dynamic modification. Trypsin was indicated as the proteolytic enzyme with two missed cleavages. Peptide and fragment mass tolerance were set at ±1.6 and 0.6 Da and precursor mass range of 350–5000 Da, and peptide charges were set excluding +1. SEQUEST results were filtered with the target PSM validator to improve the sensitivity and accuracy of the peptide identification. Using a decoy search strategy, target false discovery rates for peptide identification of all searches were <1% with at least two peptides per proteins, and the results were strictly filtered by ΔCn (<0.01), Xcorr (≥1.5) for peptides, and peptide spectral matches (PSMs) with high confidence, that is, with *q-*value of ≤0.05. Proteins quantification were conducted using the total spectrum count of identified proteins. Whereas, additional criteria were applied to increase confidence that PSMs must be present in all three biological replicates samples. Normalization of identified PSMs among LC-MS/MS runs were done dividing individual PSMs of proteins with total PSMs and average of % PSM count was utilized for calculating fold changes for different treatments. For contrasting relative intensities of proteins among control, LPS, Statin-LPS, and statin groups, samples were evaluated using cumulative confident normalized PSM values.

### Bioinformatics analysis

Protein encoding genes were functionally categorized based on gene ontology classification such as biological processes, molecular activity, and cellular components by the PANTHER gene classification system^56^. Protein abundance were visualized as a heat map, and hierarchical clustering was applied with complete linkage based on Pearson distance metrics. The heat map was generated by MeV software (ver. 4.9; http://www.tm4.org/)^57^. The proteins interactions, pathways, upstream regulatory analysis, and functional networks were generated through the use of IPA (QIAGEN Inc., https://www.qiagenbio-informatics.com/products/ingenuity-pathway-analysis)^34^. Fold changes of the commonly identified proteins were calculated by comparing the abundances among the treated and control samples (Table S2A). The proteomic data set, which included UniProt identifiers and fold changes of total identified proteins, was submitted into Ingenuity Pathway Analysis (IPA) for core analysis (Ingenuity Systems, Redwood City, CA). The matched proteins encoding genes from Ingenuity Knowledge Base generated molecular networks according to biological as well as molecular functions. These includes canonical pathways, upstream regulatory analysis, and disease-based functional networks, which helped discovering the list of biomarkers. The core analysis was carried out with the settings of indirect and direct relationships between focused molecules based on experimentally observed data, and mouse databases in the Ingenuity Knowledge Base were considered as the data sources in those analyses^58^. Right-tailed Fisher’s exact test was utilized to determine the probability that biological function and/or disease was involved with proteins. Networks were also enriched utilizing candidate proteins, namely plectin (Plec)- and prohibitin 2.

### Immunoblotting and immunocytochemistry

For immunoblotting, cells were washed with PBS twice and then lysed with RIPA buffer (same as protein preparation). Protein samples were prepared in 2X sodium dodecyl sulfate (SDS)-sample buffers and heated for 5 min at 95 °C. Proteins were separated on a 12% sodium dodecyl sulfate- polyacrylamide gel electrophoresis (SDS- PAGE) and were transferred to a 0.45 µm nitrocellulose membrane for 1.5 hr at 100 V. The nitrocellulose membrane was blocked in solution of skim milk (5%) in a Tris-buffer saline-Tween 20 (TBST) buffer for 2 hr at room temperature (RT) and then incubated with primary antibodies against plectin (ab32528; Abcam) and prohibitin 2 (SC-67045; Santa Cruz) in skim milk (5%) at 4 °C for overnight. Goat anti-rabbit IgG was used as a secondary antibody conjugated to HRP (Abcam) for 2 hr at RT. The anti-GAPDH was used as a loading control. The targeted protein band was visualized using a Clarity™ western enhanced chemiluminescent substrate (BioRad, California).

To cross-validate data on plectin protein on macrophages and BMDM cells, cells were grown on 1 M HCl treated slides cover slip in 6-well petri dish, and then fixed with chilled methanol for 5 min at RT. Subsequently, the cells were permeabilized with 0.1% Triton X-100 in PBS for 10 min. The cells were blocked with bovine serum albumin and glycine in PBS for 30 min at RT under dark condition; after that, they were incubated with anti-plectin (ab83497, Abcam) at 4 °C overnight under dark condition. They were then incubated with the secondary antibody (goat anti-rabbit IgG H&L, Alexa Flour 488, ab15007, Abcam) for 2 hr under dark condition at RT. The probed cells were viewed under confocal microscope (Zeiss LSM 510).

To examine the re-arrangement of filamentous-actin (F-actin) upon the treatment of LPS and statin at Raw 264.7 macrophage cells with or without plectin knockdown, cells were grown on 1 M HCl-treated glass slides. They were then fixed with 4% paraformaldehyde in phosphate buffer saline (PBS; non-cold) for 10 min and were subsequently permeabilized with 1% Triton X-100 in PBS for 5 min. The cells were stained with Phalloidin-iFluor 488 CytoPainter (ab176753; abcam) at room temperature in a dark condition for 60 min. Cell nuclei were labeled with propium iodide for 5 min. The stained cells were viewed under confocal microscope (Zeiss LSM 510 Meta).

### Transfection with Plectin siRNA

Plectin-targeted small interfering RNAs (siRNA; SR421444) and nonsense siRNA were purchased from OriGene (OriGene, MD, USA). For transfections, Raw 264.7 macrophage cells (4 × 10^5^ cells) were seeded in 6 well plate with DMEM medium with supplement of 10% FBS and 1% penicillin/streptomycin. After 50~70% confluency, cells were transfected with siRNA of plectin and universal scramble (negative control) at the final concentration of 10 nM according the manufacturer instructions. After 48 hr of transfections, cells were treated with statin for 24 hr and LPS for 1 hr. The cells were collected for mRNA analysis for confirming the siRNA knockdown efficiency. Total RNA from scramble and siRNA treated samples were extracted by Trizol methods. Total RNA extraction and qRT-PCR methods were described in detail in the supplemental information. Optimization results were shown in Fig. S5. LPS and statin-treated cells with/without transfections were stained with Phalloidin-iFluor 488 for imaging the filamentous-actin cytoskeleton (see immunocytochemistry section).

### Statistical analysis

The data are shown in the graph with a mean ± standard error (SEM). Statistical significance was determined using one sample t-test. A value of *P* ≤ 0.05 was considered as significant. The GraphPad Prism, version 6, was used for statistical analysis (GraphPad Software, Inc).

### Data availability

The datasets generated during and/or analyzed during the current study are available from the corresponding author on reasonable request.

## Electronic supplementary material

Supplementary Information

Table 1

Table 2
