# Supplementary material for: Inflammatory Proteomic Network Analysis of Statin-treated and Lipopolysaccharide-activated Macrophages
Source: Sci Rep. 2018 Jan 9;8:164. doi: 10.1038/s41598-017-18533-1 (PMC5760528; doi:10.1038/s41598-017-18533-1)
Supplement: Supplementary file 1 — Supplementary Information [file 41598_2017_18533_MOESM1_ESM.pdf]

Inflammatory Proteomic Network Analysis of Statin-treated and Lipopolysaccharide-activated Macrophages

Abu Hena M. Kamal[1], Jayanta K. Chakrabarty[1], S.M. Nashir Udden[2], Md. Hasan Zaki[2], Saiful M. Chowdhury[1]*

[1]Department of Chemistry and Biochemistry, University of Texas at Arlington, Texas 76019, USA
[2]Department of Pathology, UT Southwestern Medical Center, Dallas, TX 75390, USA

Supplemental Information

Corresponding author*

Saiful M. Chowdhury, PhD
Assistant Professor
Chemistry and Biochemistry
University of Texas at Arlington
PO Box 19065
700 Planetarium Place, Room 130
Arlington, TX 76019
schowd@uta.edu
Tel: 817-272-5439
Fax: 817-272-3808

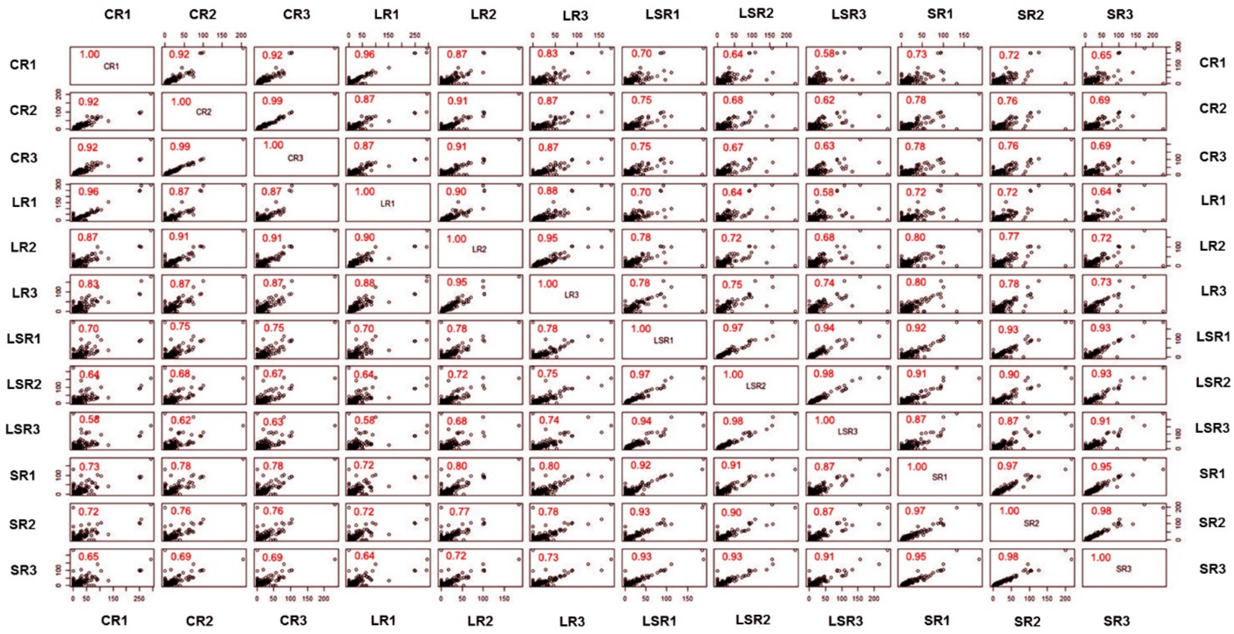

Fig. S1. Scatter plots and pair wise correlations show significant correlation patterns among the replicate samples with treatments of LPS and statin in Raw 264.7 macrophage cells. The PSMs (spectral counts) of proteins of replicate samples are plotted against each and every protein on the *x*-axis and *y*-axis, correspondingly. Every spot symbolizes the abundance of a protein, and corresponds to Pearson's correlation coefficient (R^2) of 1. The scatter plot and pair wise graph was generated by R package ver. 3.3.1. Indications: R; biological replication, C; control, L; LPS, and S; statin.

Plectin and prohibitin 2 based network by LPS stimulation

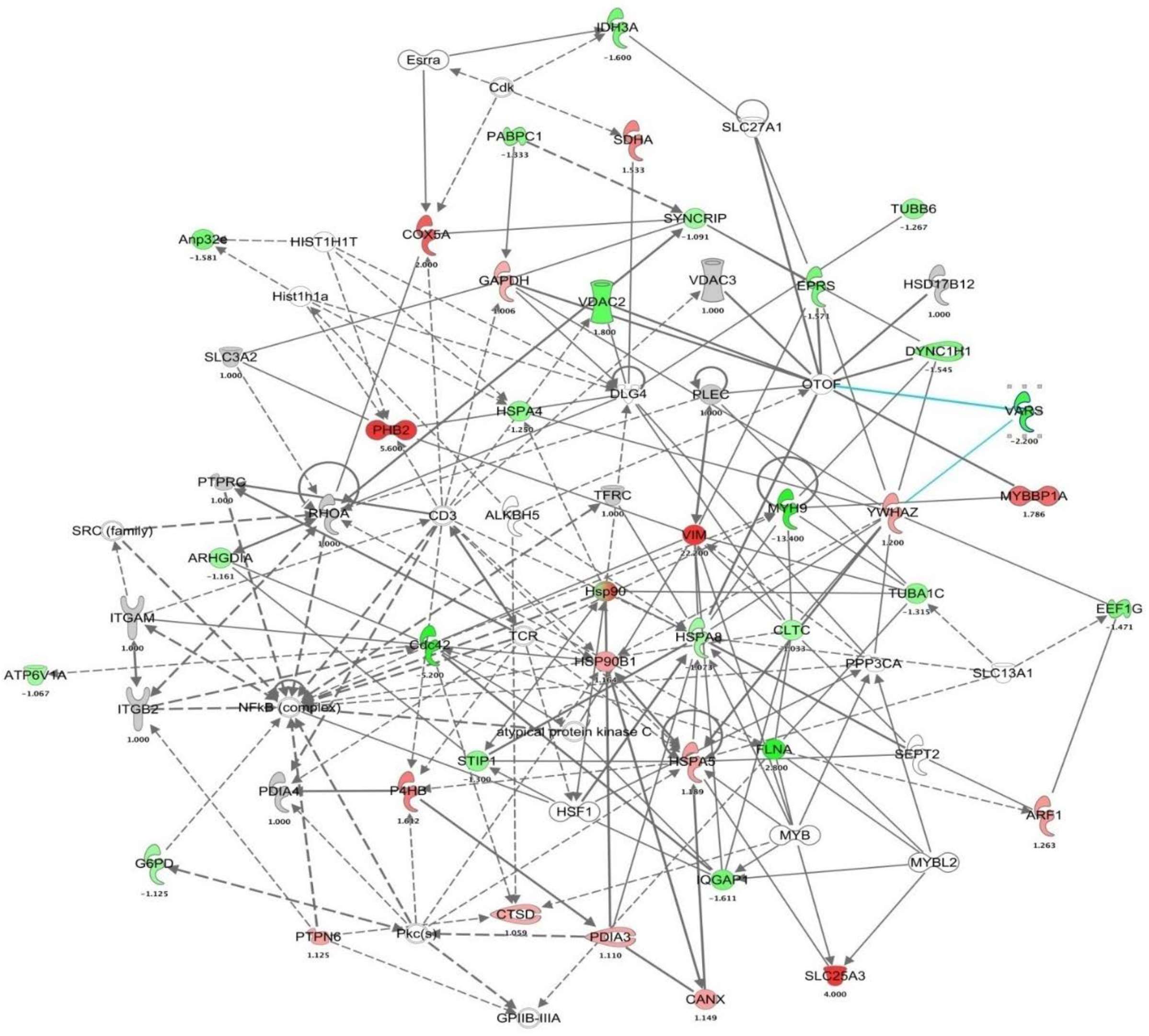

Plectin and prohibitin 2 based network at the treatment of Statin-LPS

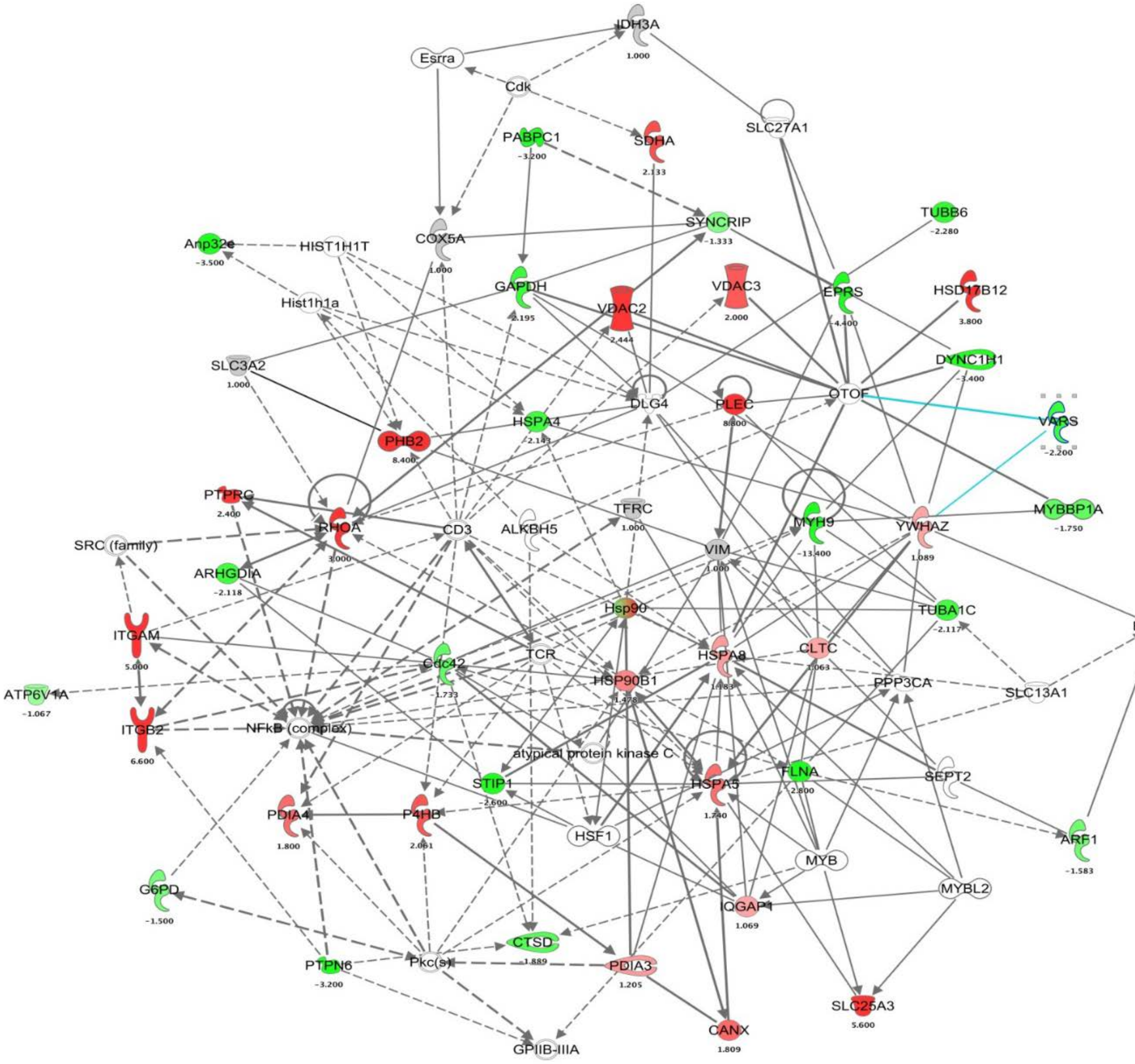

Fig. S2. IPA based plectin and prohibitin 2 proteins network in Raw 264.7 macrophage cells at the treatment of LPS and statin-LPS. The proteins interactions networks were generated through the use of IPA (QIAGEN Inc., https://www.qiagenbio-informatics.com/products/ingenuity-pathway-analysis, Reference- Kramer, A., Green, J., Pollard, J. J. & Tugendreich, S. Causal analysis approaches in Ingenuity Pathway Analysis. *Bioinformatics* **30,** 523–530 (2014).

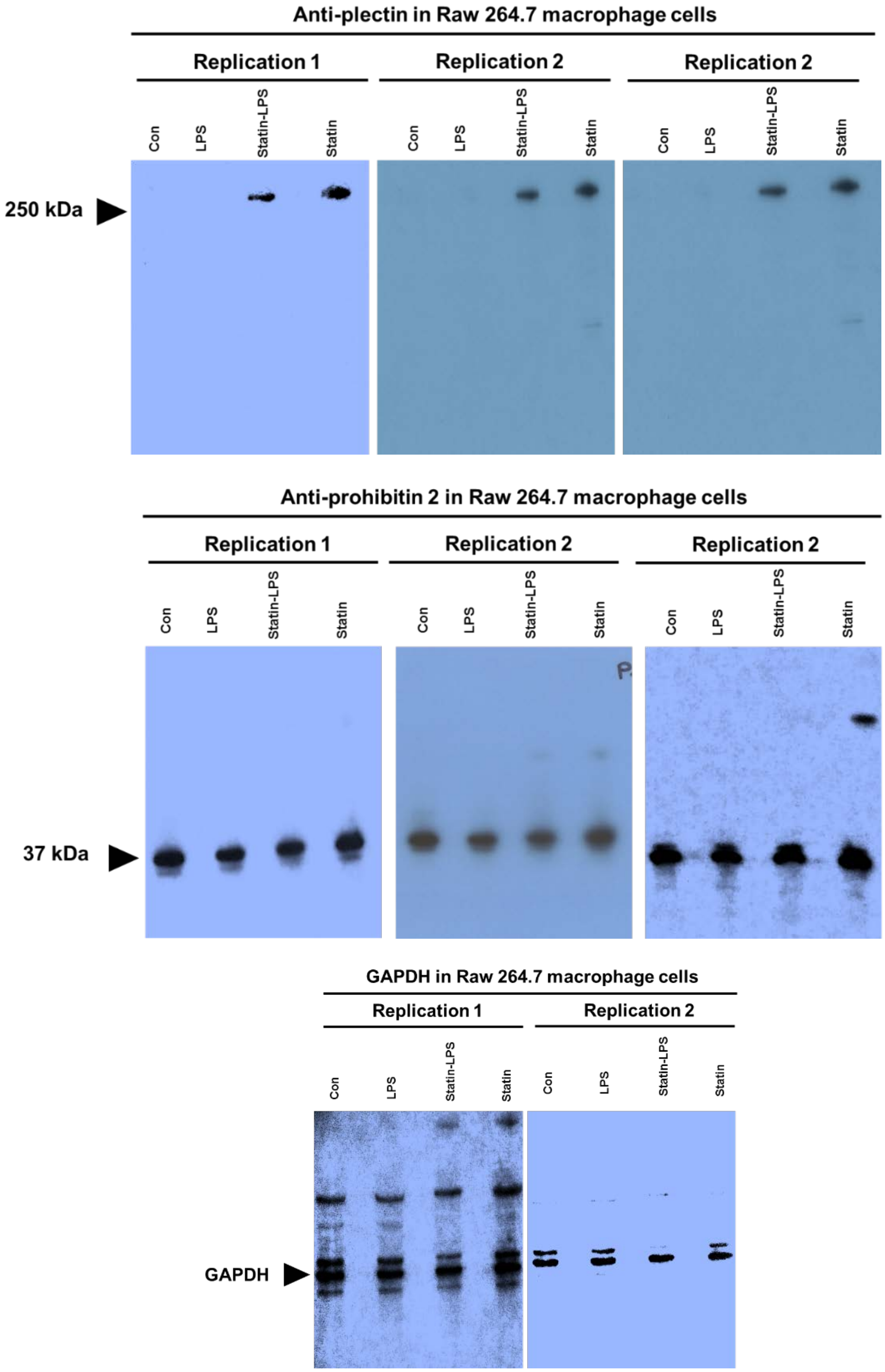

Fig. S3. Raw data of anti-plectin, anti-prohibitin 2, and GAPDH western blot in Raw 264.7 macrophage cells upon the treatment of LPS and statin.

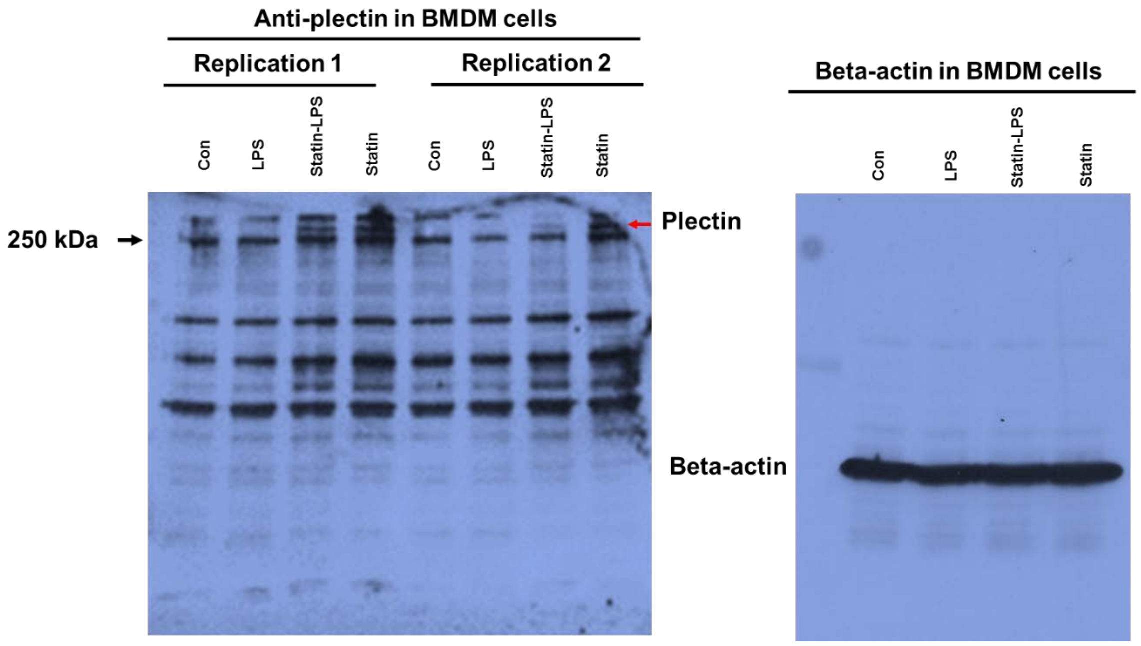

Fig. S4. Raw data of anti-plectin and beta-actin western blot in BMDM cells upon the treatment of LPS and statin.

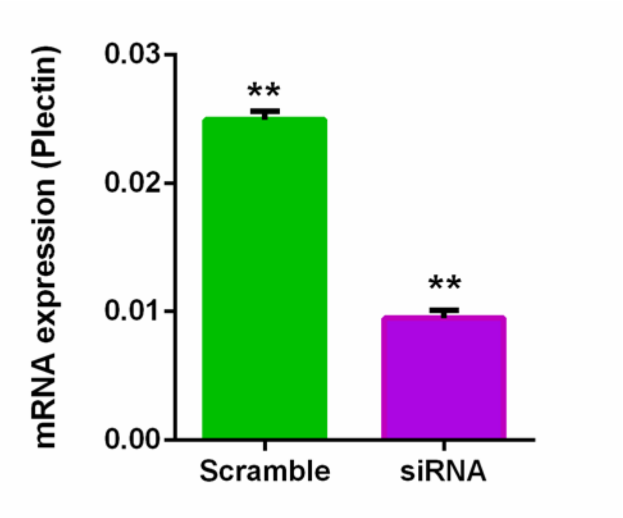

Fig. S5. Optimizing the siRNA experiments in Raw 264.7 macrophage cells. Universal scramble was used as negative control for optimizing the siRNA efficiency.

Total RNA extraction and real-time PCR

Total RNA was isolated from cultured cells with TRIzol® (Invitrogen). First-strand cDNA synthesis was performed using one-step cDNA synthesis kit (Origene, MD, USA). Real-time PCRs were performed on the CFX96 real-time system (Bio-Rad) using the SsoAdvanced™ Universal SYBR® Green Supermix (Bio-Rad). Each assay was performed in triplicate, and the mean value was used to calculate the mRNA expression for the gene of interest and the housekeeping reference gene (GAPDH). The amount of the gene of interest in each sample was normalized to that of the reference control using the comparative (2^−ΔCT) method following the manufacturer's instructions. Synthesized primers (MP222552; Plec1 and MP205604; GAPDH) were purchased from OriGene (OriGene, MD, USA).

Table legends:

Table S1. List of identified proteins from replicate samples in Raw 264.7 macrophage cells based on the treatment of LPS and statin. List were generated using proteins commonly shared among control, LPS, Statin-LPS, and Statin (A), shared among any of three treatments (B), and exclusively identified proteins in each treatments (C). Mean and standard deviation (SD) values were calculated using normalized % PSMs from three biologically replicates.

Table S2. List of identified proteins with their expression in LPS-stimulated and statin-treated Raw 264.7 macrophage cells. Lists were generated using proteins which were commonly shared among control, LPS, Statin-LPS, and Statin (A), shared among any of three treatments (B), and exclusively identified proteins in each treatments (C).
